# Effectiveness of Computed Tomography in the Diagnosis of Novel Coronavirus-2019

**DOI:** 10.7759/cureus.8134

**Published:** 2020-05-15

**Authors:** Isil Yurdaisik

**Affiliations:** 1 Radiology, Istinye University Gaziosmanpasa Medical Park Hospital, Istanbul, TUR

**Keywords:** novel coronavirus, coronavirus disease, computed tomography, covid-2019

## Abstract

Coronaviruses (CoV) belong to the coronavirus genus of the coronaviridae family. All CoVs are pleomorphic RNA viruses containing crown-like peplomers of 80-160 nm in size. This virus is a zoonotic pathogen seen with a wide range of clinical features from asymptomatic state to intensive care in humans.

So far, seven human coronaviruses have been identified with the last one being Coronavirus-2019 (COVID-19). These pathogens typically lead to mild disease, but SARS and MERS type coronaviruses have caused severe respiratory disease and even mortality within the last 20 years.

COVID-19 virus has rapidly spread worldwide after China and is continuing to cause huge economical and social impacts. Given the scarcity of resources including healthcare staff, hospital capacities, test kits, etc., timely diagnosis and treatment of this virus are of paramount importance. However, there is no vaccination or drug developed for the treatment of this disease up to today. Because the spreading rate of the virus is very high worldwide and there is no definitive treatment, diagnosis becomes even more important.

The objective of this review is to evaluate the use of chest computed tomography, one of the commonly used radiologic imaging modalities, in the diagnosis of COVID-19 in light with the current literatüre.

## Introduction and background

A series of pneumonia cases with unknown causes have been reported from the Wuhan state of China. A few days later, this mysterious causative agent of pneumonia was declared as a novel coronavirus [1]. After severe respiratory syndrome coronavirus (SARS-CoV) in 2002 and Middle East respiratory syndrome coronavirus (MERS-CoV) in 2012, novel coronavirus (COVID-19) was introduced in the human population as a highly pathogenic and large-scale epidemic coronavirus [2]. This novel virus was previously named as severe acute respiratory syndrome (SARS) coronavirus-2, and later the virus was named as COVID-19 by the World Health Organization (WHO). On January 20, 2020, the WHO officially declared that COVID-19 became an epidemic as an international public health emergency. On March 11, 2020, COVID-19 was characterized as a pandemic by the WHO [3]. As of April 10, 2020, 622,251 confirmed COVID-19 cases and 46,430 deaths were reported in Europe region [4].

Coronaviruses (CoV) belong to the coronavirus genus of the coronaviridae family. All CoVs are pleomorphic RNA viruses containing crown-like peplomers of 80-160 nm in size. This virus is a zoonotic pathogen seen with a wide range of clinical features from an asymptomatic state to intensive care in humans. Although most coronaviruses affect animals, these are zoonotic pathogens that can be transmitted between animals and humans. So far seven human coronaviruses have been identified with the last one being COVID-19. These pathogens typically lead to mild disease, but SARS and MERS type coronaviruses have caused severe respiratory disease and even mortality within the last 20 years. Today, the COVID-19 virus has rapidly spread worldwide after China and is continuing to cause huge economic and social impacts. Given the scarcity of resources including healthcare staff, hospital capacities, test kits, etc., timely diagnosis and treatment of this virus are of paramount importance. There is no vaccination or drug developed for the treatment of this disease up to today. Because the spreading rate of the virus is very high worldwide and there is no definitive treatment, the diagnosis becomes even more important [5].

The objective of this review is to evaluate the use of chest computed tomography (CT), one of the commonly used radiologic imaging modalities, in the diagnosis of COVID-19 in light with the current literature.

## Review

Epidemiology

COVID-19 was identified in China for the first time, but it has rapidly spreaded all over the world in a short time and currently is increasingly reported in all continents. The number of cases outside China outpaced the number and rate of new cases in China [6]. Epidemiological studies have reported that the outbreak was associated with wild animals sold in a seafood market in Wuhan province [7]. However, later confirmed cases without a history of exposure to this market indicated human-to-human transmission of COVID-19. As the epidemic progressed, human-to-human transmission has become the main transmission route of the virus.

All ages are sensitive to COVID-19. However, the virus is more fatal in the elderly population. The infection is transmitted through droplets produced during cough and sneezing [8]. According to recent studies in the literature, viral loads are higher in the nasal cavity compared to the throat; thus, there is no significant difference between symptomatic and asymptomatic people [9]. Infected droplets can spread a few meters, accumulating on surfaces. COVID-19 can survive for days under positive atmospheric conditions, although it can be destroyed in shorter than one minute using common disinfectants such as sodium hypochlorite and hydrogen peroxide. The infection occurs by inhalation of these droplets or touching the mouth, nose, and eyes with the hands after touching contaminated surfaces. 

Clinical features

The incubation period of COVID-19 is thought to be within 14 days after the exposure. However, the majority of cases have been seen within 4-5 days of exposure [10]. In a study including 1099 patients with a confirmed diagnosis of COVID-19, median incubation duration was found as four days [11]. Clinical features of COVID-19 vary from asymptomatic state to acute respiratory distress syndrome and multiorgan dysfunction. Common clinical manifestations include fever (not in all cases), cough, sore throat, headache, and shortness of breath. In a study, fever was reported as 83%, cough as 82%, and shortness of breath as 31% of COVID-19 patients [12]. However, there are still no specific clinical features to reliably distinguish COVID-19 from other viral respiratory infections. In a part of patients, the disease may progress to pneumonia, respiratory failure, and death at the end of the first week. According to a study from China, around 15% of the patients presented with severe symptoms and 5% required intensive care [13]. In Italy and Spain, 7% to 12% of the hospitalized patients were reported to require intensive care [14].

Diagnosis

The first approach in COVID-19 is to early detect suspected cases, immediately isolate these people, and implement infection control measures. The diagnosis of COVID-19 is established with fever, cough, and/or dyspnea together with a history of contact with an infected person in a distance shorter than approximately 2 meters and/or travel to the areas, where the disease is common within the last 14 days. However, COVID-cases may be asymptomatic and even may not have a fever. This disease is confirmed with positive molecular testing. According to the American Centers for Disease Control and Prevention (CDC), test priority criteria in suspected COVID-19 patients that were updated as of March 8, 2020 are as follows [15]:

Hospitalized patients with signs and symptoms compatible with COVID-19.

Elderly people (≥ 65 years) and people with chronic medical conditions and/or immunocompromised status (e.g. diabetes mellitus, heart disease, using immunosuppressive medications, chronic lung disease, chronic kidney disease) who can be at risk for a poor outcome.

Including healthcare personnel, people with a history of close contact with other persons with suspected or laboratory-approved COVID-19 or travel to the affected geographic areas up to 14 days before the onset of symptoms.

Molecular tests used for the diagnosis of COVID-19 are performed on respiratory samples (throat swab, nasopharyngeal swab, sputum, and endotracheal aspirates). The virus can also be identified in stool and blood in severe cases. The diagnosis of COVID-19 is mainly established with reverse transcription-polymerase chain reaction (RT-PCR) test. However, this test requires strict laboratory specifications and the results take a long time [16]. Other laboratory investigations are usually non-specific. Indicators such as white blood cells, platelets, C-reactive protein (CRP), and erythrocyte sedimentation rate (ESR), alanine aminotransferase/aspartate aminotransferase (ALT/AST), prothrombin time, creatinine, D-dimer, and low-density cholesterol (LDL) are also studied. Nevertheless, these markers have been associated with disease severity and are not specific to COVID-19 [17].

Chest X-ray is one of the diagnostic modalities used to help the diagnosis of COVID-19. This image usually shows bilateral infiltrates. However, the results of this method can be normal at the beginning of the disease. Chest CT is more sensitive and specific in the diagnosis of COVID-19 compared to X-ray. CT images usually show infiltrates, ground-glass opacities (GGO), and sub-segmental consolidation. In addition, CT is abnormal also in asymptomatic patients without clinical findings related to the involvement of the lower respiratory tract. CT scans have been used also in suspected COVID-19 patients with a negative molecular diagnosis, and repeated molecular tests were positive in most of these patients [17].

CT in the diagnosis of COVID-19

Imaging plays an important role in the diagnosis and management of COVID-19. Chest CT is used as the first-line imaging method in suspected cases and is a useful tool for monitoring the changes during treatment. Correct diagnosis of viral pneumonia based on chest CT indicates isolation and plays an important role in the management of patients suspected to have an infection, especially in the absence of scientifically proven treatment methods. Findings on CT may reflect the severity of the disease. Therefore, CT is an important imaging technique in helping the diagnosis and management of COVID-19 patients, and publications about the radiologic appearance of COVID-19 pneumonia are increasing in the literature. As this disease increasingly becomes a global concern, it is more crucial for radiologists to be familiar with CT images of COVID-19 and to have basic knowledge about the clinical course and size of this infection. Radiologists to have sufficient knowledge about the clinic and chest CT imaging of COVID-19 will help early detection of the infection and evaluation of the disease course.

So far reported common CT features regarding COVID-19 are as follows [18]:

● GGO

● Consolidation

● Mixed GGO and consolidation

● Centrilobular nodules

● Architectural distortion

● Cavitation

● Tree-in-bud

● Bronchial wall thickening

● Reticulation

● Subpleural bands

● Traction bronchiectasis

● Intrathoracic lymph node enlargement

● Vascular growth in the lesion

● Pleural effusions

CT images are usually examined in four distributions as craniocaudal, transverse, lung region, and scattered. Thin slice chest CT is more effective in the early detection of COVID-19 [19]. Some case series and case reports investigating chest CT imaging features of COVID-19 have been published [20-23]. It has been reported that CT abnormalities are more likely to be bilateral, to have a peripheral distribution, and to involve the lower lobes [24]. In a study comparing 219 COVID-19 patients in China and 205 patients with other viral pneumonia in the USA, peripheral distribution, GGOs, thin reticular opacities, vascular thickening, and reverse halo sign were more common, while central and peripheral distribution, air bronchogram, pleural thickening, pleural effusion, and lymphadenopathy were more rare in COVID-19 cases [25].

In a study from Wuhan, China, it was reported that COVID-19 had abnormal findings on chest CT even in asymptomatic patients [26]. In that study, chest CT findings of 81 patients with COVID-19 were examined. Examinations were performed by two radiologists who were blind to RT-PCR testing results. According to the results of the study, CT images showed bilateral lung involvement in 79%, peripheral distribution in 54%, and diffuse distribution in 44% of the patients. The most common chest CT findings included GGO together with poorly defined margins, smooth or irregular interlobular septal thickening, air bronchogram, and thickening of adjacent pleura [26]. Remarkably, in a study by Shi et al., early CT changes were seen in the asymptomatic patient group, and these findings supported the observations in a familial cluster with COVID-19 pneumonia [19]. However, some studies have reported positive RT-PCR results in the absence of CT changes or abnormal CT results in the presence of initial false-negative RT-PCR results [18]. Predominant detection of GGOs on CT in COVID-19 makes this method more sensitive. In a study by Song et al., the most common chest CT findings were reported as GGO appearance in COVID-19 patients [21]. In a case series of 21 patients by Chung et al., GGO was the most common CT finding by 57%, and bilateral disease was found in 76% of COVID-19 patients [27]. However, CT findings may be normal also in confirmed cases [28].

In a study by radiologists from Wuhan, China, it was found that chest CT had a low misdiagnosis rate in COVID-19 and this method can help standardization of imaging and a rapid diagnosis [10]. On the other hand, it has been reported that the use of CT is limited in the detection of specific viruses and making a distinction between viruses [29]. In a study from Wuhan including 1014 patients who underwent RT-PCR testing and chest CT, the sensitivity of positive results by the consensus of two radiologists was found as 97% when PCR tests were used as a reference. However, in the same study, the specificity of CT was only 25% [30]. It was reported that this low specificity may be associated with other etiologies leading to CT findings. In general, chest findings of chest CT in COVID-19 overlap with the findings of other infections such as H1N1, SARS, and MERS, and this limits the specificity of CT.

Effectiveness of CT in COVID-19 should be carefully evaluated because the majority of the existing data come from the studies conducted in the far east. According to the studies in the literature, the preference of CT in China is resulted from the wide availability of this method, because access to CT is relatively easier in China [31]. In most of these studies, it was reported that chest CT was positive in the presence of negative test results [17]. RT-PCR results can be affected by sampling errors and low viral load [32]. In previous studies about SARS, the sensitivity of RT-PCR has been shown to be insufficient within the first five days of the disease [33]. In addition, findings of this rapidly spreading conditions have not been completely published or are not updated. CDC currently does not recommend the use of CT for the diagnosis of COVID-19. According to the CDC, viral testing is the only specific diagnosis method, and even in the case of COVID-19 radiologic findings with CT, the results must be confirmed with viral testing [34]. According to Lee et al. who are radiologists in Hong Kong university, the variability of COVID-19 presentations leads to difficulties in establishing the diagnosis. The authors emphasized that there are more things to learn from this contagious viral pneumonia, and further studies are needed on the relationship between CT findings and clinical progression [31].

**Figure 1:**
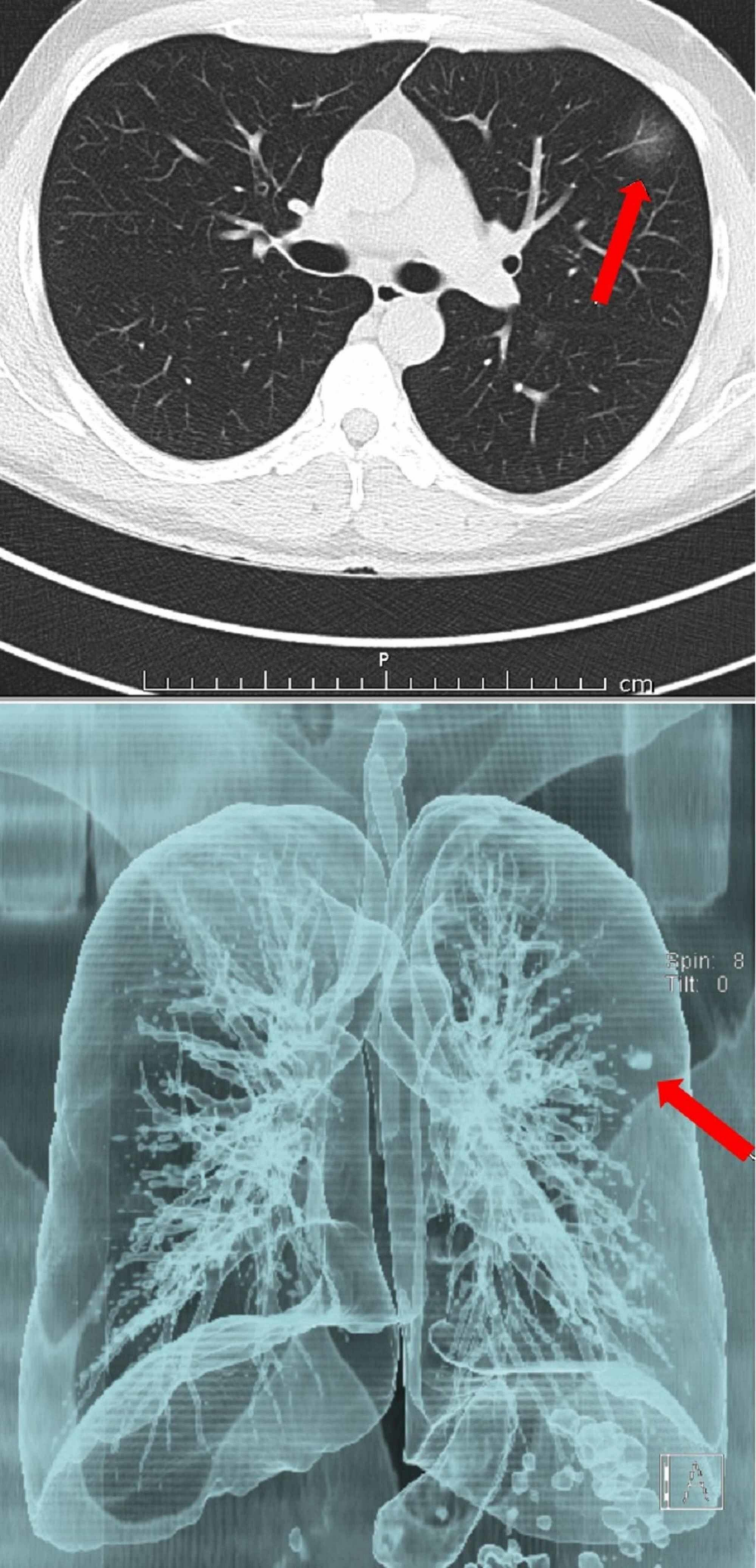
A 39-year-old male patient: PCR (+) for COVID-19 Upper left and peripheral localized lesion of ground-glass density, which is hardly noticeable on cross-sectional CT is clearly seen on 3D reconstruction. CT, computed tomography; PCR, polymerase chain reaction

**Figure 2:**
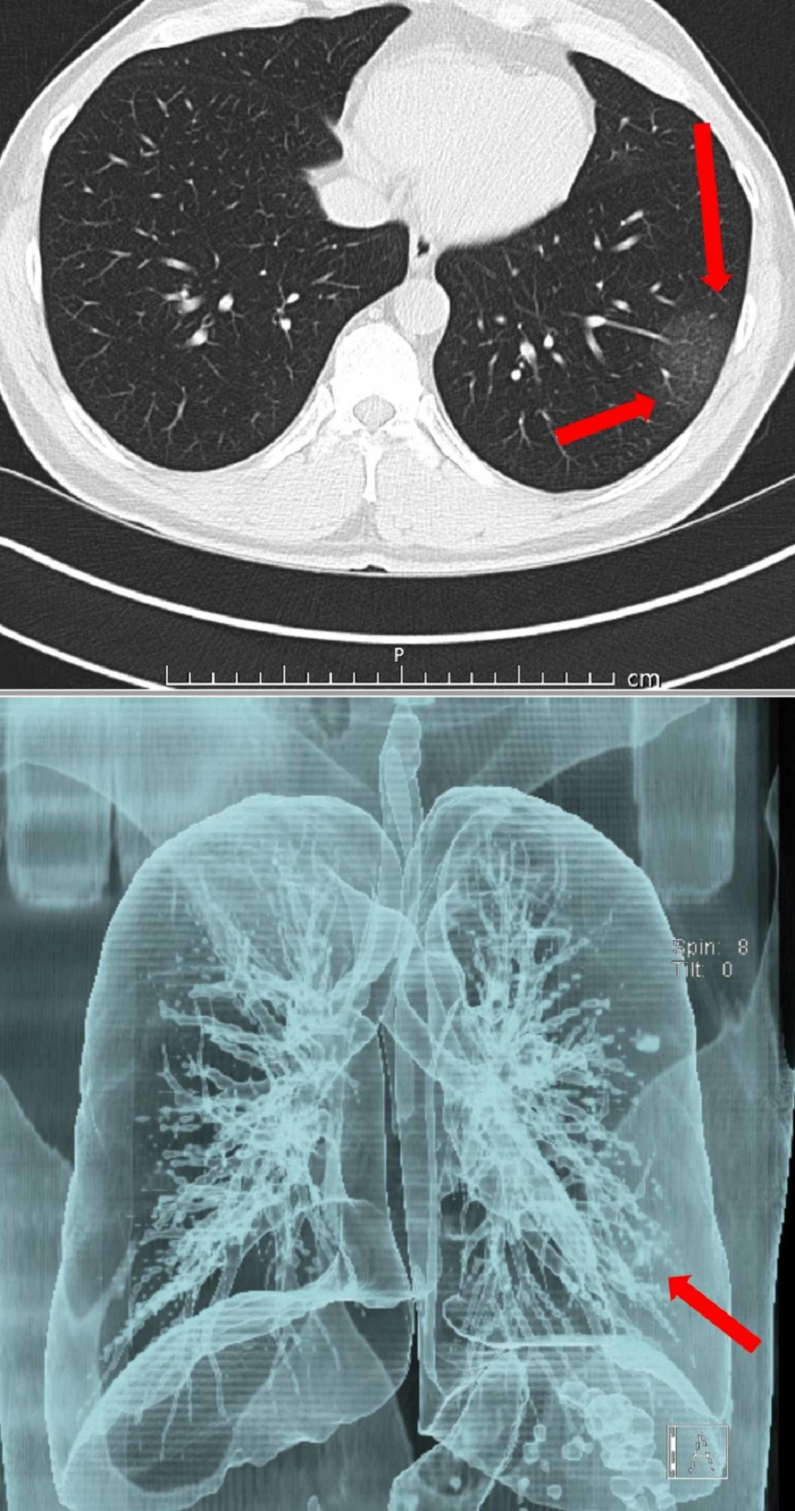
At the same examination, a lesion of gross ground density in the lower left lobe is well visualized on the sectional view, but it cannot be distinguished in the bronchovascularization on 3D reconstruction

**Figure 3:**
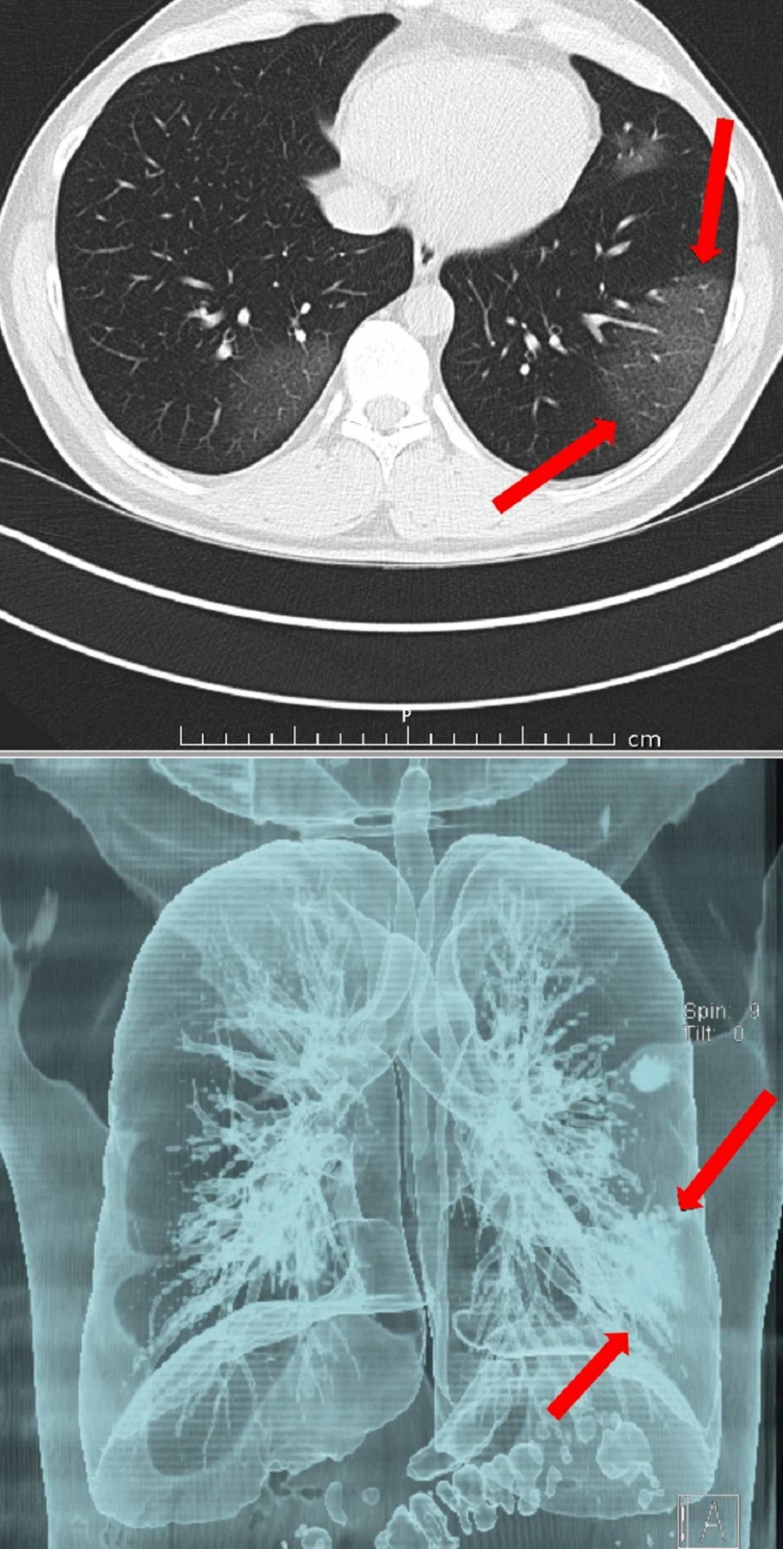
CT examination of the same patient 2 days later The upper left lesion is enlarged and the lower left lesion is visible. CT, computed tomography

**Figure 4:**
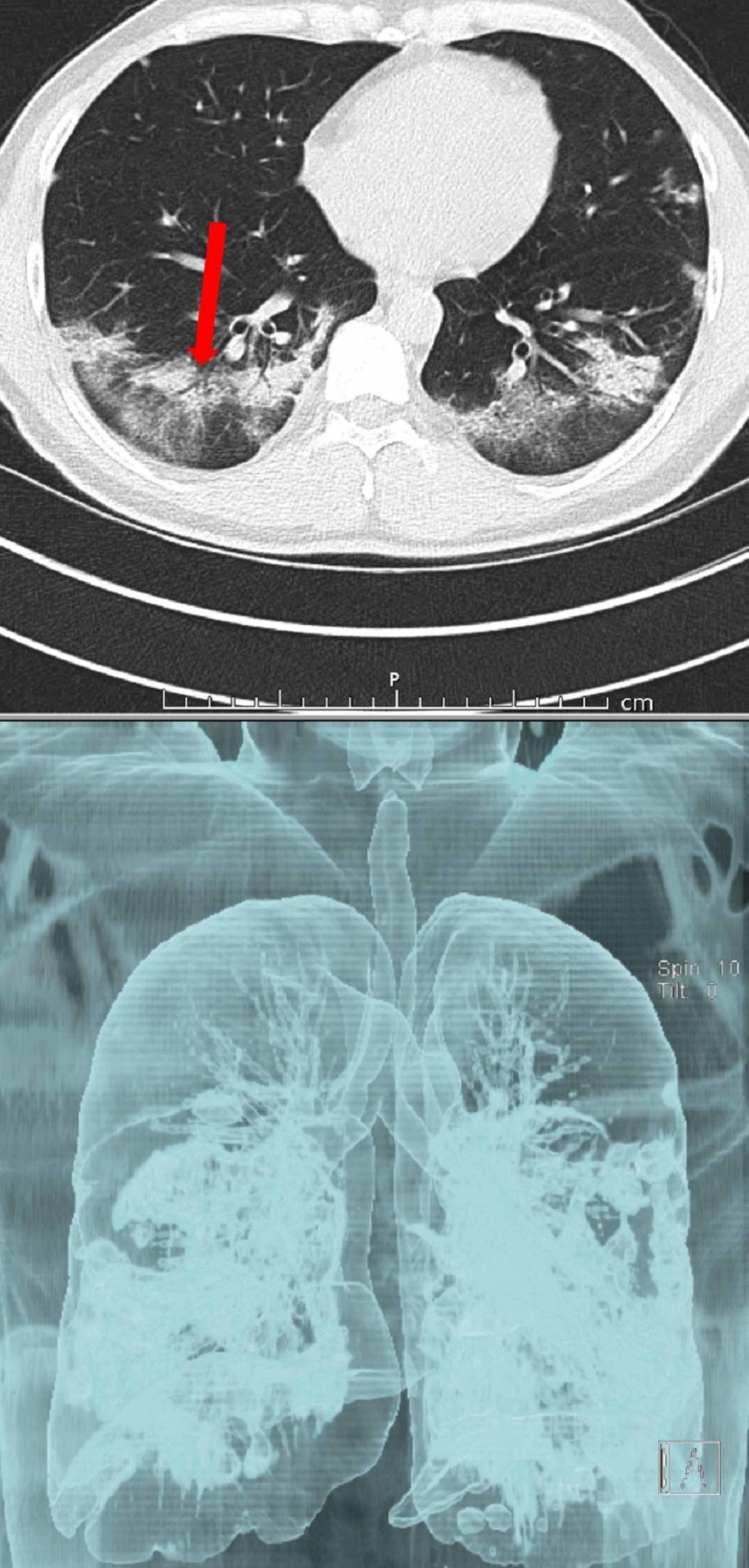
CT examination of the same patients 12 days later Air bronchograms and fibrosis were developed. Bilateral milimetric central aeration areas on 3D image. Sectional routine image better shows lesion details, and 3D images better depict involvement area in the lung. CT, computed tomography

CT scans of a patient with COVID-19 are shown in Figures 1-4.

## Conclusions

There is debate in the literature on the use of chest CT for the diagnosis of COVID-19. As this epidemic progresses, various presentations of COVID-19 will be increasingly observed, and the correlation between CT and RT-PCR findings will be more commonly studied. As the picture becomes clearer, the radiologic-pathologic correlation will be better understood, potential imaging predictors will be determined in more detail, and the role of radiology in the management of COVID-19 will be increased.
